# Remote team-based learning during COVID-19: lower academic performance is associated with the exclusion of test grades from final grades

**DOI:** 10.31744/einstein_journal/2025AO1007

**Published:** 2025-01-24

**Authors:** Juliana Magdalon, Leandro Luongo Matos, Marcelo Vivolo Aun, Marcelo Madeira, Welbert de Oliveira Pereira, Durval Anibal Daniel, Elda Maria Stafuzza Gonçalves Pires

**Affiliations:** 1 Hospital Israelita Albert Einstein Faculdade Israelita de Ciências da Saúde Albert Einstein São Paulo SP Brazil Faculdade Israelita de Ciências da Saúde Albert Einstein, Hospital Israelita Albert Einstein, São Paulo, SP, Brazil.

**Keywords:** Team-based learning, Academic performance, School, medical, Students, medical, Learning, Problem-based learning, Educational measurement, Group process

## Abstract

**Introduction:**

The COVID-19 pandemic necessitated a shift from in-person to remote learning, including team-based learning activities. Many studies have explored several aspects of team-based learning; however, there is still a lack of knowledge regarding the factors affecting students’ attitudes and performance.

**Objective:**

To evaluate the impact of the COVID-19 pandemic on medical students’ performance in individual readiness assurance tests during team-based learning activities, given that their grades were excluded from the final course grades.

**Methods:**

We compared the number of team-based learning applications, student absences, and individual readiness assurance tests grades of medical undergraduate students in three different scenarios: Group 1, in-person individual readiness assurance tests before the pandemic; Group 2, remote individual readiness assurance tests during the lockdown period when their grades were excluded from the final grade calculation; and Group 3, in-person individual readiness assurance tests during the pandemic after lockdown restrictions eased.

**Results:**

Remote team-based learning led to significantly lower grades than in-person evaluations. Moreover, the number of students with failing or insufficient grades (below seven) increased during the remote learning period, when their grades were excluded from the final grade calculation, compared to in-person individual readiness assurance tests before the pandemic. Furthermore, absenteeism was higher when the online strategy was applied.

**Conclusion:**

We observed lower academic performance by medical students during remote team-based learning sessions, likely owing to the exclusion of individual readiness assurance tests grades from the final course grade calculations during the COVID-19 pandemic.

## In Brief

Magdalon et al. demonstrated that excluding iRAT grades from the final grade calculation of the course negatively impacted student performance and attendance in team-based learning sessions.

## Highlights

■ Remote team-based learning sessions were conducted during the COVID-19 pandemic, and their grades were not included in the final course grade calculation.
■ Remote team-based learning resulted in lower iRAT grades than in-person team-based learning, independent of the semester.
■ Remote team-based learning resulted in more students failing the test than in-person team-based learning.
■ Remote team-based learning resulted in lower student attendance than in-person team-based learning.

## Graphical abstract

**Figure:**
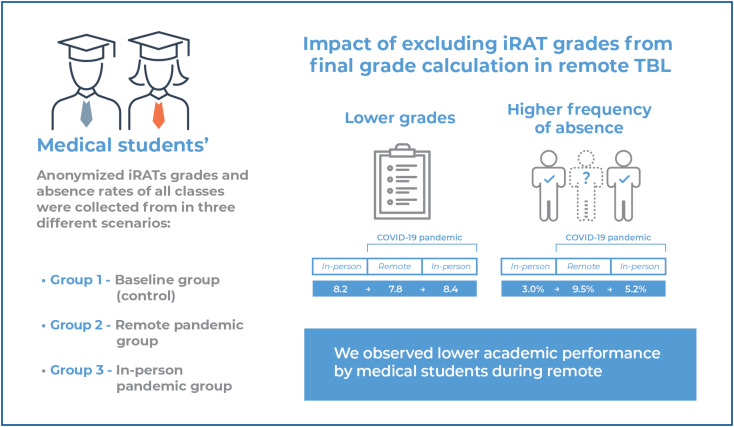

## INTRODUCTION

The COVID-19 pandemic forced all in-person classes and assessments to occur remotely. This transition has been termed emergency remote teaching to distinguish it from existing online learning courses.^(1)^ This shift severely impacted assessment, which had to be conducted online using available technology.^(2)^ The lack of online proctoring posed a particular challenge to all educational institutions in maintaining academic integrity during assessments. To mitigate cheating risks, many institutions adopted open-book examinations, although studies have reported significant grade differences compared with traditional closed-book examinations.^(3,4)^

Team-based learning (TBL) is an active learning method that focuses on achieving specific learning objectives in an environment in which students are held responsible for both individual and group learning. In TBL, teachers assign students to teams and present them with challenging problems that require collaborative problem-solving. Students are expected to attend the online class prepared and equipped with the necessary knowledge to answer group application questions.^(5)^ The use of TBL in health education has increased over recent decades. Michaelsen's conceptual model for TBL addresses several elements that may impact learner engagement and learning outcomes, such as teacher decisions, individual characteristics, contextual factors, and team characteristics.^(5)^ A systematic review has shown that most studies in this area focus on professor and student attitudes, as well as learning outcomes. Most of these studies show that TBL results in higher satisfaction than traditional classes, concluding that this is an effective instructional technique. However, several aspects of TBL require further investigation, including learner engagement and factors affecting academic outcomes.^(6)^

During the COVID-19 pandemic, TBL faced several challenges, including technological limitations (including Internet access), reduced interaction, and difficulty monitoring student engagement. Therefore, educators shared strategies for successful online TBL adaptations.^(7-10)^ However, concerns about academic integrity in graded activities, such as individual and team readiness assurance tests (iRAT and tRAT), remain significant, especially with the use of open-book tests undermining the objectives of TBL.

The medical school investigated in this study continued to use TBL as a learning strategy during emergency remote teaching but decided not to factor iRAT scores into the final grade owing to concerns about academic integrity. This provided an opportunity to explore gaps in the literature regarding some dimensions of TBL, particularly the lack of information regarding TBL performance during the COVID-19 pandemic. The primary aim of this study was to evaluate the impact of emergency remote teaching during the COVID-19 pandemic on iRAT grades during TBL activities. We compared the iRAT grades across three scenarios: 1) in-person iRATs before the pandemic, 2) remote iRATs during the lockdown period when their grades were excluded from the final course grade, and 3) in-person iRATs during the pandemic after lockdown restrictions were eased.

## OBJECTIVE

To evaluate the impact of the COVID-19 pandemic on medical students’ individual readiness assurance tests grades during team-based learning activities, specifically in the context where these grades were excluded from their final course grades.

## METHODS

### Setting

This study was performed at the medical school *Faculdade Israelita de Ciências da Saúde Albert Einstein*, Brazil, where TBL is employed as the main learning strategy. Team-based learning occurred in all courses over the first seven semesters, two to three times per week. The remaining five semesters were dedicated to internships. Each class consisted of 60 students, who were randomly divided into nine groups of six to seven students. At the beginning of each semester, the groups were re-divided.

Team-based learning at the institution follows all core elements.^(11)^ In the preparatory phase, the teacher provides learning goals and assigns materials that students must study individually, such as book chapters, videos, and articles. In class, learning is assessed through an iRAT, followed by a tRAT after a consensus-building discussion. Both tests are identical and consist of approximately ten multiple-choice questions about the most important concepts from prior studies. During the tRAT, students receive immediate feedback if the answer is correct. If an incorrect answer is assigned, they have another chance to identify a new consensus answer, which is repeated until the correct answer is detected, thus decreasing the final grade of each question. Subsequently, the teacher clarifies the students’ doubts about the previous tests and rapidly begins the application phase, in which students are presented with different scenarios and problems to be solved. They must discuss the answer in the same group as in the tRAT, make a specific choice, and display it simultaneously with the other groups. The teacher promotes debates between groups, and students must explain their choices. Student participation is peer evaluated and counted as part of the TBL grade together with the other phases of the activity (iRAT counts for 20%, tRAT counts for 30%, and peer assessment counts for 50% of the TBL grade).

Both iRAT and tRAT are performed through the web-based learning platform Canvas^®^ (Instructure^®^ Inc., Salt Lake City, Utah, USA), regardless of whether the class is in person or remote. Each discipline includes two to 13 TBL sessions during each semester, along with other active learning methodologies, depending on the number of hours dedicated to that discipline every week.

From March 16^th^, 2020, until the end of the semester (June 2020), every activity in the first seven semesters (before the internship) was performed online and synchronously, including TBL sessions. After an academic break in July 2020, some in-person activities resumed in August 2020, with social distancing measures in place. During this period, some TBL sessions were held in person (split between two rooms with two teachers), although most continued online. When TBL was conducted remotely, students still received iRAT and tRAT grades, but they did not count toward the final course grade and were used solely for performance evaluation. In contrast, when TBL was conducted in person, even during the COVID-19 pandemic, iRAT and tRAT grades were included in the final course grades, influencing students’ pass or fail outcomes.

### Data collection

To evaluate the effects of the COVID-19 pandemic on TBL activities, the anonymized iRAT grades and absence rates from all classes were collected from the Canvas^®^ platform across three different scenarios:

Group 1 - Baseline group (control): This group included data from the first and second semesters of 2019, as well as the first semester of 2020 before the official start of the COVID-19 lockdown in Brazil (March 16^th^, 2020), when the iRATs were performed in person.

Group 2 - Remote pandemic group: This group included data from the first semester of 2020 during the lockdown in Brazil (starting March 16^th^, 2020) and the second semester of 2020, when iRATs were performed exclusively remotely and not included in the final course grades (online by Zoom Meeting^®^ Tool; Zoom Video Communications, San Jose, CA, USA).

Group 3 - In-person pandemic group: This group included data from the second semester of 2020 and the first semester of 2021, during the COVID-19 pandemic, when iRATs were performed in person but with social distancing measures in place (activities were split between two rooms with two teachers).

This study was approved by the Institutional Research Ethics Committee of *Hospital Israelita Albert Einstein* (CAAE: 49031821.6.0000.0071; # 5.363.350).

### Statistical analyses

All collected data were analyzed and expressed as means with corresponding standard deviations (SDs), standard errors (SEs), medians, interquartile ranges, minimum and maximum values, and 95% confidence intervals (95%CI). Group comparisons of means were performed using one-way analysis of variance (ANOVA) followed by Bonferroni's post-hoc test. Categorical comparisons between groups were analyzed using the χ^2^ test. All statistical analyses were performed using SPSS^®^ version 27.0 (IBM^®^ Inc., New York, USA), and statistical significance was set at p<0.05.

## RESULTS

During the 30-months study period, 842 TBL sessions were conducted, yielding 41,849 iRAT grades. In 2019, 381 in-person TBL sessions took place across two semesters. During the first semester of 2020, at the beginning of the COVID-19 pandemic in Brazil, 153 TBL sessions were conducted, 65.4% of which were conducted online. In the second semester of 2020, 151 TBL sessions occurred, of which 72.8% were held online. By the first semester of 2021, students had engaged in 157 TBL sessions, with 63.7% conducted online. Following the easing of lockdown restrictions (second semester of 2020), 98 iRATs were performed using a new in-person strategy (Group 3), with students divided into two rooms with two teachers to maintain social distance. Descriptive data for all TBL sessions and comparisons between the different strategies are presented in table 1.

**Table 1:** Descriptive data and comparisons between team-based learning and absences across periods of the year, course semesters and rotations for different groups (basic–between 1^st^ and 4^th^ semesters; clinical–between 5^th^ and 7^th^ semester), according to each different scenario (Group)

Comparison		TBL distribution				p value*	Absences distribution				p value*
		Group 1	Group 2	Group 3	Total		Group 1	Group 2	Group 3	Total	
Period of the year											
	1^st^ semester 2019	194 (100.0)	0 (0.0)	0 (0.0)	194 (100.0)	0.001	300 (3.1)	--	--	300 (3.1)	0.001
	2^nd^ semester 2019	187 (100.0)	0 (0.0)	0 (0.0)	187 (100.0)		221 (2.3)	--	--	221 (2.3)	
	1^st^ semester 2020	53 (34.6)	100 (65.4)†	0 (0.0)	153 (100.0)		130 (4.6)	482 (9.1)†	--	612 (7.5)	
	2^nd^ semester 2020	0 (0.0)	110 (72.8)†	41 (27.2)	151 (100.0)		--	429 (7.2)†	86 (3.9)	515 (6.3)	
	1^st^ semester 2021	0 (0.0)	100 (63.7)†	57 (36.3)	157 (100.0)		--	716 (12.3)†	193 (6.1)	909 (10.1)	
Course period											
	1^st^ semester	61 (55.5)	42 (38.2)	7 (6.4)	110 (100.0)	0.117	163 (4.7)	328 (12.2)†	79 (16.7)	570 (8.6)	0.001
	2^nd^ semester	62 (57.4)	41 (38.0)	5 (4.6)	108 (100.0)		50 (1.6)	212 (8.5)†	16 (5.4)	278 (4.6)	
	3^rd^ semester	68 (53.5)	43 (33.9)	16 (12.6)	127 (100.0)		133 (3.9)	286 (1.5)	35 (3.6)	454 (6.6)	
	4^th^ semester	59 (47.2)	52 (41.6)	14 (11.2)	125 (100.0)		132 (4.4)	308 (10.8)†	56 (6.9)	496 (7.5)	
	5^th^ semester	61 (48.8)	44 (35.2)	20 (16.0)	125 (100.0)		79 (2.6)	156 (7.1)†	20 (2.0)	255 (4.1)	
	6^th^ semester	59 (47.6)	49 (39.5)	16 (12.9)	124 (100.0)		32 (1.1)	183 (7.5)†	25 (3.2)	240 (4.0)	
	7^th^ semester	64 (52.0)	39 (31.7)	20 (16.3)	123 (100.0)		62 (2.0)	154 (7.8)†	48 (4.7)	264 (4.4)	
Rotation											
	Basic	250 (53.2)	178 (37.9)	42 (8.9)‡	470 (100.0)	0.002	478 (3.7)	1,134 (10.8)†	186 (7.2)	1,798 (6.9)	0.001
	Clinical	184 (49.5)	132 (35.5)	56 (15.1)	372 (100.0)		173 (1.9)	493 (7.5)†	93 (3.3)	759 (4.1)	
	Total	434 (51.5)	310 (36.8)	98 (11.6)	842 (100.0)	-	651 (3.0)	1,627 (9.5)	279 (5.2)	2,557 (5.8)	-

* χ^2^ test;

† Significant higher frequencies, demonstrated by the p value;

‡ Significant lower frequency, demonstrated by the p value.

Note: Group 1–Baseline group (control): data from the first and second semesters of 2019, as well as first semester of 2020, before the official start of lockdown period of the COVID-19 pandemic in Brazil (March 16th, 2020), when the iRATs were performed in-person; Group 2–Remote pandemic group: data from the first semester of 2020 during the lockdown period of the COVID-19 pandemic in Brazil (started on March 16th, 2020), and second semester of 2020 when iRATs were performed exclusively remotely (online); Group 3–In-person pandemic group: data from the second semester of 2020 and first semester of 2021, during the COVID-19 pandemic, in cases where the iRATs were performed in-person but with social distance measures in place (activity performed in two rooms with two professors).

**Figure 1:**
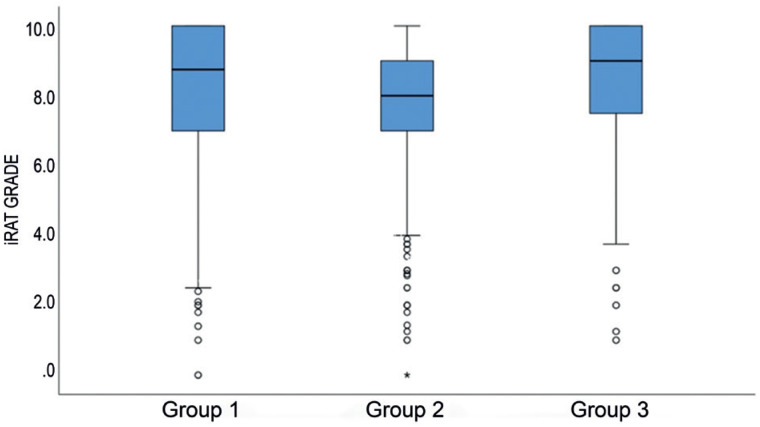
Boxplot charts demonstrating lower grades for iRAT (p<0.001, one-way ANOVA) for the online remote strategy (Group 2), when grades were excluded from the final course grade, comparing to in-person team-based learning before the COVID-19 pandemic (Group 1) and in-person team-based learning during the pandemic after the lockdown period (Group 3)

The remote TBL strategy (Group 2) resulted in significantly lower grades (7.826; 95%CI= 7.796-7.856; p<0.001; one-way ANOVA) when compared to in-person evaluations, where TBL grades contributed to the final course grades, regardless of whether they were assessed before or during the COVID-19 pandemic (8.225; 95%CI = 8.203-8.247 for the baseline period [Group 1] and 8.464; 95%CI = 8.420-8.508 for the period after the lockdown [Group 3]; Table 2 and Figure 1). Moreover, the iRAT grades were significantly higher after the lockdown period than in the control group (p<0.001; Bonferroni's post-hoc test, one-way ANOVA). The remote iRAT grades were lower regardless of the semester or phase (basic or clinical), as shown in table 3.

**Table 2:** Means differences between iRAT grades, according to each different scenario (Group)

	n	Mean	SD	95% CI		SE	Minimum value	Maximum value
				Low	High			
Group 1	21,233	8.225	1.629	8.203	8.247	0.011	0.0	10.0
Group 2	15,520	7.826	1.917	7.796	7.856	0.015	0.0	10.0
Group 3	5,096	8.464	1.600	8.420	8.508	0.022	1.0	10.0
Total	41,849	8.106	1.753	8.089	8.123	0.008	0.0	10.0

p<0.001, one-way ANOVA (p<0.001 for each pairwise comparison; Bonferroni's post-hoc test).

SD: standard-deviation; SE: standard-error.

Note: Group 1–Baseline group (control): data from the first and second semesters of 2019, as well as first semester of 2020, before the official start of lockdown period of the COVID-19 pandemic in Brazil (March 16th, 2020), when the iRATs were performed in-person; Group 2–Remote pandemic group: data from the first semester of 2020 during the lockdown period of the COVID-19 pandemic in Brazil (started on March 16th, 2020), and second semester of 2020 when iRATs were performed exclusively remotely (online); Group 3–In-person pandemic group: data from the second semester of 2020 and first semester of 2021, during the COVID-19 pandemic, in cases where the iRATs were performed in-person but with social distance (activity performed in two rooms with two professors).

**Table 3:** Comparisons between iRATs grades and period of the year, course period (semester) and rotation (basic: between 1^st^ and 4^th^ semesters; clinical: between 5^th^ and 7^th^ semester), according to each scenario (Group)

Comparison		n	Mean	SD	SE	Minimum value	Maximum value	p value*
1^st^ semester								
	Group 1	3,290	8.284	1.538	0.027	0.0	10.0	<0.001
	Group 2	2,356	7.815‡	1.793	0.037	0.0	10.0	
	Group 3	395	8.084	1.541	0.077	3.0	10.0	
2^nd^ semester								
	Group 1	3,143	8.138	1.596	0.028	0.0	10.0	<0.001
	Group 2	2,294	7.624‡	1.892	0.039	0.0	10.0	
	Group 3	282	8.794	1.291	0.077	4.0	10.0	
3^rd^ semester								
	Group 1	3,254	8.122	1.656	0.029	0.0	10.0	<0.001
	Group 2	2,198	7.414‡	1.995	0.042	0.0	10.0	
	Group 3	949	8.128	1.769	0.057	1.3	10.0	
4^th^ semester								
	Group 1	2,838	8.524	1.534	0.029	0.0	10.0	<0.001
	Group 2	2,552	8.131‡	1.825	0.036	0.0	10.0	
	Group 3	756	8.636	1.372	0.050	3.0	10.0	
5^th^ semester								
	Group 1	2,975	8.374	1.704	0.031	0.0	10.0	<0.001
	Group 2	2,046	8.116‡	1.908	0.042	1.0	10.0	
	Group 3	972	8.719	1.472	0.047	2.5	10.0	
6^th^ semester								
	Group 1	2,755	8.010	1.621	0.031	0.0	10.0	<0.001
	Group 2	2,253	7.869‡	1.927	0.041	0.0	10.0	
	Group 3	762	8.335	1.674	0.061	2.0	10.0	
7^th^ semester								
	Group 1	2,978	8.129	1.698	0.031	0.0	10.0	<0.001
	Group 2	1,821	7.783‡	2.003	0.047	1.0	10.0	
	Group 3	980	8.563	16.641	0.053	1.0	10.0	
Basic rotation								
	Group 1	12,525	8.260	1.591	0.014	0.0	10.0	<0.001
	Group 2	9,400	7.760‡	1.893	0.019	0.0	10.0	
	Group 3	2382	8.361	1.585	0.032	1.3	10.0	
Clinical rotation								
	Group 1	8,708	8.175	1.683	0.018	0.0	10.0	<0.001
	Group 2	6,120	7.926‡	1.948	0.025	0.0	10.0	
	Group 3	2,714	8.555	1.608	0.031	1.0	10.0	

* One-way ANOVA;

‡ Significantly lower mean, demonstrated by p value.

Note: Group 1–Baseline group (control): data from the first and second semesters of 2019, as well as first semester of 2020, before the official start of lockdown period of the COVID-19 pandemic in Brazil (March 16th, 2020), when the iRATs were performed in-person; Group 2–Remote pandemic group: data from the first semester of 2020 during the lockdown period of the COVID-19 pandemic in Brazil (started on March 16th, 2020), and second semester of 2020 when iRATs were performed exclusively remotely (online); Group 3–In-person pandemic group: data from the second semester of 2020 and first semester of 2021, during the COVID-19 pandemic, in cases where the iRATs were performed in-person but with social distance (activity performed in two rooms with two professors).

**Figure 2:**
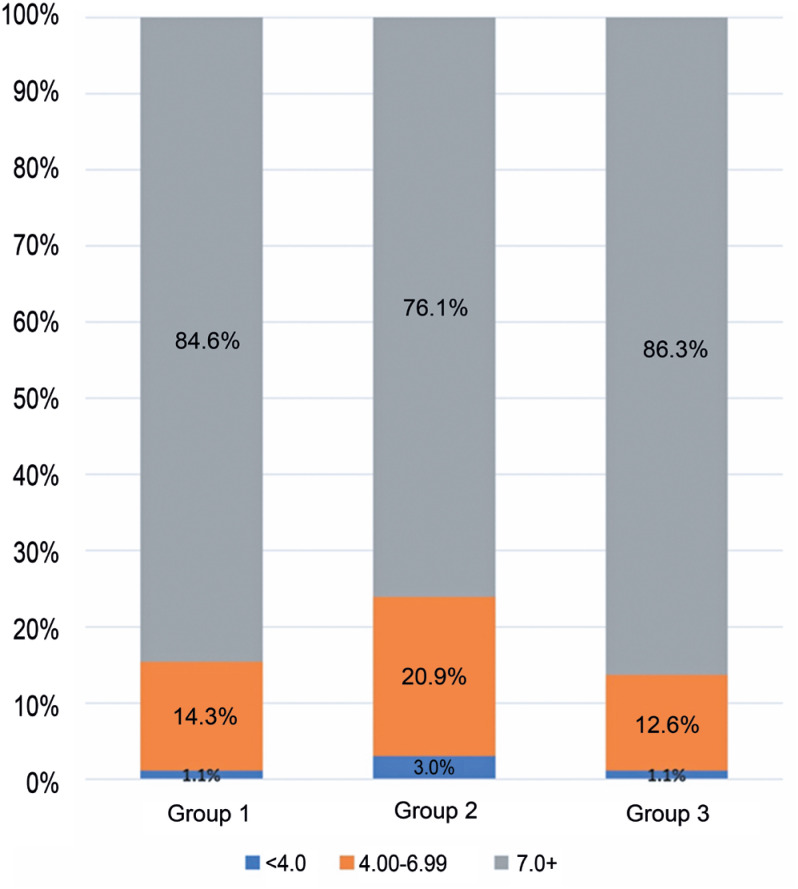
Bar chart demonstrating increased rates of students failing (orange bars) or those with insufficient grades (blue bars) during the iRAT conducted under the remote team-based learning strategy (Group 2), when grades did not contribute to final course approval

To demonstrate the impact of lower iRAT grades from remote TBL sessions, which did not count toward the final grades, we categorized these grades into three different groups, as shown in table 4 and figure 2: (1) <4.0, indicating the students who failed the test; (2) ≥7.0, representing students who passed the test; (3) 4.00-6.99, the group of students with insufficient grades. The number of students who failed the test or had insufficient grades increased from 15.4% in the baseline period (Group 1) to 23.9% when the online strategy was adopted (Group 2), and returned to 13.7% after the lockdown period (Group 3).

**Table 4:** Differences between categorized values of iRAT grades, according to each different scenario (Group)

TBL (iRAT grade)	Grade				p value*
	<4.0 n (%)	4.00-6.99 n (%)	≥7.0 n (%)	Total n (%)	
Group 1	225 (1.1)	3,041 (14.3)	17,967 (84.6)	21,233 (100.0)	<0.001
Group 2	471 (3.0)†	3,240 (20.9)†	11,809 (76.1)	15,520 (100.0)	
Group 3	55 (1.1)	641 (12.6)	4,400 (86.3)	5,096 (100.0)	
Total	751 (1.8)	6,922 (16.5)	34,176 (81.7)	41,849 (100.0)	-

* χ^2^ test;

† Significantly higher frequencies, as demonstrated by p values.

TBL: team-based learning.

Note: Group 1–Baseline group (control): data from the first and second semesters of 2019, as well as first semester of 2020, before the official start of lockdown period of the COVID-19 pandemic in Brazil (March 16th, 2020), when the iRATs were performed in-person; Group 2–Remote pandemic group: data from the first semester of 2020 during the lockdown period of the COVID-19 pandemic in Brazil (started on March 16th, 2020), and second semester of 2020 when iRATs were performed exclusively remotely (online); Group 3–In-person pandemic group: data from the second semester of 2020 and first semester of 2021, during the COVID-19 pandemic, in cases where the iRATs were performed in-person but with social distance (activity performed in two rooms with two professors).

The frequency of remote TBL sessions (Group 2), which did not count toward final grades, was not significantly affected by the semester of the medical course (p=0.117, χ^2^ test) or the phase (basic or clinical) (Table 1). This indicates that the occurrence of failing grades was widespread and affected the entire student population. After he lockdown period, fewer in-person TBL sessions occurred in the basic phase (Group 3) (Table 1; p=0.002, χ^2^ test). This decline was attributed solely to the increased number of teachers at higher risk for COVID-19, preventing the adoption of in-person strategies for those classes. Furthermore, absenteeism in TBL sessions was higher during the online strategy, where grades did not count for final approval (Table 1; 9.5% for Group 2 *versus* 3.0% and 5.2% for Groups 1 and 3, respectively; p=0.001, χ^2^ test). These absence rates were not affected by the time of year, period of the medical course, or phase.

## DISCUSSION

The COVID-19 pandemic has presented several challenges and necessitated adaptations to the learning environment, leading to a shift toward online classes and assessments.^(12)^ The aim of this study was to evaluate the impact of the COVID-19 pandemic on medical students’ iRAT grades in TBL activities conducted remotely during emergency remote teaching. Our results show that the remote TBL strategy-and particularly the fact that these grades did not count toward final course approval-resulted in significantly lower grades and a higher incidence of failing or insufficient grades compared to in-person evaluations or when grades contributed to the final grade. The frequency of remote TBL sessions was unaffected by the semester or phase of the medical course, suggesting that the occurrence of failing grades was widespread and affected the entire student cohort. In addition to the lower grades, more students were absent during iRATs in the remote TBL sessions compared to the in-person pre- and post-pandemic periods. This suggests that students were less engaged in the remote learning environment and did not adequately prepare for classes as requested by the teachers. Moreover, the exclusion of grades from final course grades likely contributed to lower academic performance, making this a key confounding factor in the results.

These findings align with existing literature indicating that grading individual performance enhances student engagement with the content and encourages pre-class preparation.^(11)^ A recent systematic review comparing face-to-face and remote TBL found no clear differences between the two approaches,^(13)^ underscoring the importance of studies like the present one in assessing various strategies for TBL application in terms of academic achievement, learning, and satisfaction. However, we cannot exclude the possibility that the low grade identified in our study stemmed from the remote learning environment, as other authors have reported similar challenges during emergency remote teaching, including loss of interest, difficulty concentrating and understanding the content, reduced interactivity and technological issues.^(14-21)^ Other possible explanation for the increased number of absences in iRATs during remote learning could be poor internet connectivity and technological barriers, although it was uncommon for students in the study to report such issues. Nonetheless, some students reported that they appreciated online learning during the pandemic, especially because of enhanced flexibility and more time for sleep or self-care^(22,23)^ although students felt that they did not prepare themselves well in advance for online learning.^(22)^

The major concern regarding students’ poor performance in online formats is their learning outcomes. The TBL iRAT phase assesses student knowledge acquired in the preparatory phase and includes questions about the most important concepts of the theme. In cases where students are not prepared for class, all subsequent phases (the tRAT and application phases) may be compromised, affecting overall student learning. Furthermore, considering that students have studied less for remote TBL sessions or that their grades were not counted for final approval, it is likely that they also performed similarly in all other remote non-TBL classes, which may have impacted the entire learning of those disciplines during the COVID-19 pandemic. During remote TBL sessions, fostering a collaborative learning environment proved more challenging than in in-person classes. Teachers relied on Zoom Meeting^®^ breakout rooms to perform tRATs and group discussions during the application phase. However, since teachers could not monitor all the group conversations simultaneously, it was common to close the breakout rooms before discussions were completed, which likely hindered deep learning opportunities for students who had prepared themselves and had higher grades on the iRAT. Deep learning often depends on opportunities for students to explain their reasoning to others.^(24)^

Several studies conducted during the COVID-19 pandemic have evaluated the effectiveness of synchronous online TBL as a valuable tool for continuing education for health professional students.^(25-27)^ These studies indicated that remote and in-person TBL was approved by students and even led to improved test performance compared to students who did not voluntarily sign up to use the TBL method. Nonetheless, we could not find any studies that specifically investigated the impact of remote TBL when grades did not affect the final course grades. While we acknowledge that TBL is one of the most effective deep-learning tools, face-to-face teaching may yield better outcomes. We believe that the iRAT and tRAT results should count toward final grades to encourage students to be well prepared during pre-class study.

Another important aspect to consider is academic integrity during remote TBL. Given that TBL grades were not factored into final course grade, most teachers did not alter questions from the previous semesters. Conversely, when TBL sessions were conducted in-person, teachers were instructed to change the questions of the iRAT/tRAT, as students had access to past tests. Although students could have consulted previous tests during remote iRATs, which might have resulted in higher grades, our findings suggest otherwise. This indicates that most students upheld academic integrity during remote TBL sessions, despite an increased perception of cheating in online assessments.^(28-30)^

### Strengths and limitations

To our knowledge, this is the first study to compare TBL grades before and during the COVID-19 pandemic, particularly in a medical school that uses TBL as its main learning strategy. One significant advantage is that all teachers and students (except for first semester students) were already proficient in the methodology, minimizing the possibility that any observed difficulties regarding remote TBL were due to a lack of knowledge of the method. Additionally, the high number of student grades analyzed provided robust data, enhancing the reliability of the results.

However, this study has some limitations. First, although we carefully collected and analyzed all data, we may have missed some data from TBL activities, particularly during remote learning, since some teachers may have used platforms other than Canvas^®^ for assessments, and only tests recorded in Canvas^®^ were considered. Second, during the study period, two courses changed faculty, which may have affected the difficulty level of the iRAT/tRAT questions. However, given the extensive dataset, we believe that such variations were isolated and did not significantly influence the overall findings. Third, the study lacked a fourth group: an in-person setting where TBL grades did not count toward the final grade. This group could have helped determine whether the lower academic achievement in remote TBL sessions was due to the remote learning environment or a lack of grade-based consequences. Unfortunately, this scenario was not included in the present study because it was not part of the medical school's educational program. Future qualitative research using student focus groups could enhance our understanding of how remote learning affects academic achievement.

## CONCLUSION

This study indicated that medical students showed lower academic performance in remote individual readiness assurance tests during the COVID-19 pandemic, possibly because the team-based learning grades did not count toward their final course grades.
